# P-2276. Shifting trends over time in patients admitted with presumed or confirmed *Pneumocystis jirovecii* pneumonia without underlying human immunodeficiency virus infection from 2006 to 2023 in Singapore

**DOI:** 10.1093/ofid/ofae631.2429

**Published:** 2025-01-29

**Authors:** Matthew C Y Koh, Jinghao Nicholas Ngiam, Paul Tambyah, Lionel H W Lum

**Affiliations:** National University Health System, Singapore, Singapore; National University Health System, Singapore, Singapore; National University Hospital, Singapore, Singapore, Not Applicable, Singapore; NUHS, Singapore, Not Applicable, Singapore

## Abstract

**Background:**

*Pneumocystis jirovecii* pneumonia (PJP) is an opportunistic infection associated with significant morbidity and mortality in immunocompromised hosts. PJP has emerged as a significant pathogen affecting non-HIV infected hosts, with wider use of novel immunosuppressive therapies. We sought to describe changing trends in the clinical profile and outcomes of patients without underlying HIV diagnosed with PJP.

Clinical characteristics of patients with pneumocystis pneumonia without underlying HIV

**Figure:**
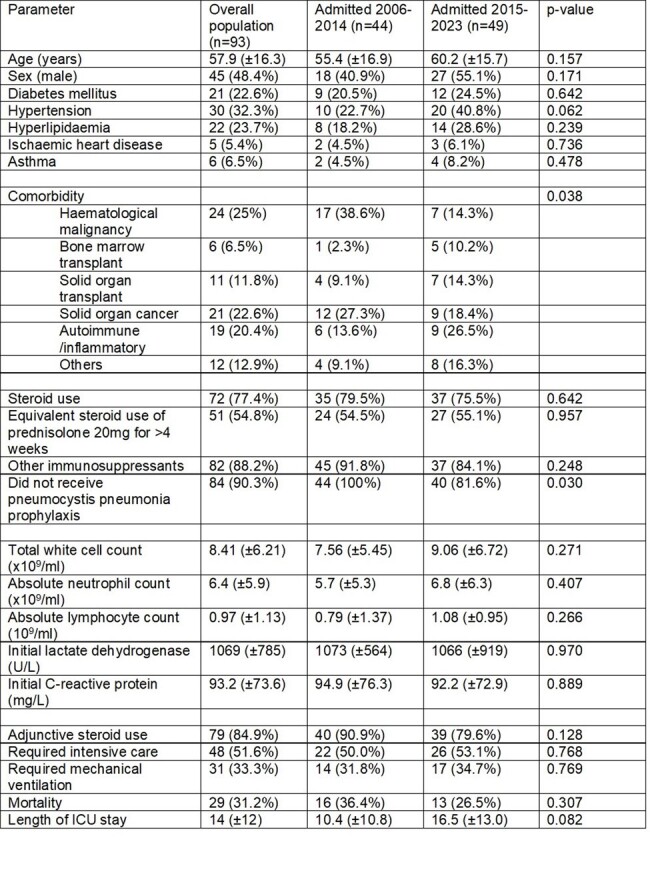

Clinical characteristics of patients with pneumocystis pneumonia without underlying HIV

**Methods:**

A total of 93 consecutive patients without HIV, with presumed or confirmed PJP, admitted to a tertiary hospital in Singapore were studied from 2006-2023. The cohort was divided into the first 44 patients from 2006-2014, and the second 49 patients from 2015-2023. Data on each patient’s clinical background, presenting symptoms, laboratory data as well as clinical outcomes such as need for intensive care, mechanical ventilation or mortality were tabulated.

**Results:**

Over the years, there was no significant differences in the age or sex of patients with PJP. However, there was a decrease in proportion of haematological malignancy as a predisposing condition for PJP (38.6% vs 14.3%), but an increase in the proportion of patients with autoimmune/inflammatory conditions presenting with PJP (13.6% vs 26.5%). A smaller proportion of patients presented without PJP prophylaxis in more recent years (100.0% vs 81.6%, p=0.030), and shortness of breath was a less common symptom (90.9% vs 63.3%, p=0.002). A greater proportion of patients were diagnosed using PJP PCR. Overall mortality (36.4% vs 26.5%), as well as the need for mechanical ventilation (31.8% vs 34.7%) and intensive care (50.0% vs 53.1%) did not differ between the groups.

**Conclusion:**

In this single centre retrospective study, there have been significant changes over almost two decades in the clinical profile of patients without underlying HIV who had PJP infection. A greater proportion of patients had autoimmune and inflammatory conditions, while the proportion of those with haematological malignancy decreased. This may be due to the implementation of standardised protocols for PJP prophylaxis. More cases were detected using PCR, which appears to be a helpful adjunct to microscopic examination. Morbidity and mortality from PJP in such non-HIV immunocompromised hosts however remains high and has not declined over the years.

**Disclosures:**

Paul Tambyah, MBBS (S'pore), Diplomate, American Board of Internal Medicine and Infectious Diseases, Moderna: Grant/Research Support|Sanofi-Pasteur: Grant/Research Support

